# The Woven EndoBridge for intracranial aneurysms: Radiological outcomes and factors influencing occlusions at 6 and 24 months

**DOI:** 10.1177/19714009221122216

**Published:** 2022-08-26

**Authors:** Kemal Alpay, Antti Lindgren, Riitta Rautio, Riitta Parkkola

**Affiliations:** 1 Department of Radiology, 60652 Turku University Hospital, Turku, Finland; 2 Department of Clinical Radiology, 60650 Kuopio University Hospital, Kuopio, Finland; 3 60652 Turku University, Turku, Finland

**Keywords:** WEB, aneurysm, endovascular treatment

## Abstract

**Purpose:**

To identify factors influencing short- and mid-term radiological outcomes of intracranial aneurysms (IAs) treated with the Woven EndoBridge (WEB).

**Methods:**

A total of 112 patients were treated for IAs with the WEB in at our institution between 2013 and 2020. Patients with 6- and/or 24-months follow-up data were included in the study. Aneurysm occlusion was evaluated using the Raymond-Roy occlusion classification (RR). RR 1 and RR 2 were considered as adequate outcomes, while RR 3 inadequate.

**Results:**

Data were available for 91 patients (56 females, 62%) at 6 months and 62 of those patients (39 females, 58%) at 24 months. The adequate occlusion (RR 1/RR 2) rate was 89% (*n* = 81/91) at the 6-months follow-up and 91% (*n* = 56/62) at the 24-months follow-up. The treatment-related morbidity rate was 4% (*n* = 4/91), and mortality rate was 1% (*n* = 1/91). The predictor for inadequate occlusion at the 6-months follow-up was the lobular shape of an aneurysm (*p* = .01). The aneurysm’s height (*p* = .02), maximal diameter (*p* = .001), width (*p* = .002), aspect ratio (*p* = .03), dome-to-neck ratio (*p* = .04), and lobular shape (*p*= .03) were predictive factors for inadequate occlusion at 24 months. All the thrombosed aneurysms (*n* = 3) showed unfavorable radiological outcomes and required re-treatment within 24 months. None of the patient-related factors were significant.

**Conclusions:**

The WEB provides favorable occlusion rates and low complications for both ruptured and unruptured wide-necked IAs. Unfavorable radiological outcomes after WEB treatment may be related to aneurysm morphology and size.

## Graphical abstract

**Graphical Abstract:**
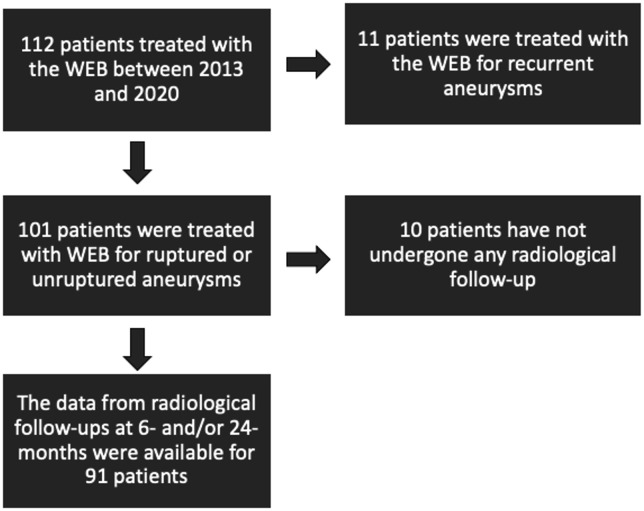

## Introduction

Endovascular treatment with simple coiling for both ruptured and unruptured intracranial aneurysms (IAs) has been proven to be safe and effective; however, the treatment of IAs with simple coiling carries a 20% risk of recanalization.^1–3^ In particular, large (≥10 mm) intracranial aneurysms with a wide neck (≥4 mm) are prone to recanalization after conventional coiling.^4^ Although stent-assisted coiling and balloon-assisted coiling provide better radiological outcomes for intracranial aneurysms than simple coiling, the rates of thromboembolic complications are non-negligible and are seen in approximately 10% of patients.^5,6^ Apart from relatively high complication rates in stent-assisted coiling, the need for dual antiplatelet medication is another disadvantage, particularly in the treatment of patients with acutely ruptured IAs.^7^ Intraluminal flow-diverters provide 90% occlusion with a low complication profile for unruptured IAs^8,9^; however, they carry high risk of complications in treatments of acutely ruptured IAs.^10^

Intrasaccular flow-diversion with the Woven EndoBridge (WEB) (Woven EndoBridge, WEB, Microvention, Tustin, CA, USA) is a treatment for wide-necked bifurcation aneurysms. The WEB is a self-expandable, retrievable, and detachable nitinol braided cage. The first-generation WEB-DL (dual layer) has been replaced with the WEB-SL (single layer) and WEB-SLS (single layer sphere).^11^ A flow quantification study showed that the WEB disturbs outflow more than inflow velocity, eventually causing thrombosis of the aneurysm.^12^ Several studies have demonstrated efficacy and safety of WEB; however, only few studies have investigated patient and aneurysms related factors influencing radiological outcomes after WEB treatment.^13–16^

The objective of our single-center retrospective study was to assess the short- and mid-term radiological outcomes of aneurysms after WEB treatment and to identify possible factors influencing the radiological outcome.

## Methods

### Data collection

The data regarding the IAs and patients (i.e., smoking status, hypertension, sex, and age) were collected from in-hospital aneurysm registry. A total of 112 patients were treated for IAs with a WEB between January 2013 and December 2020. Patients with 6- and/or 24-months follow-up data were included in the study. Patients without any follow-up data, and patients treated with the WEB for a recurrent IA were excluded. Three-Dimensional (3D) rotational angiography was used to assess the following aneurysm characteristics: the dome size, width and height, the neck size, and the origin of the parent artery. The dome-to-neck ratio (DNR) and aspect ratio (ASR) were calculated. An aneurysm considered was wide-necked if a width of the neck is ≥4 mm or the DNR was less than 2. Thrombosis in an aneurysm was assessed with computed tomography angiography or magnetic resonance angiography (MRA). An aneurysm was considered as multilobular if there were two or more daughter sacs in an aneurysm according to 3D rotational angiography.

### Neurointerventions

The aneurysms were selected for WEB treatment by neurointerventional radiologists after all conventional methods were discussed at a multidisciplinary meeting that included interventional neuroradiologists, neuroradiologists, and vascular neurosurgeons. The WEB was preferred over simple coiling, stent-assisted coiling or flow diversion mainly because of the location of the aneurysms but also because WEB does not need dual antiplatelet therapy.

All neurointerventions were carried out under general anesthesia via femoral access in a bi-plane angiographic suite. After the 3D rotational angiography images had been captured, the operator measured the dimensions of the IAs to assess the optimal size of the WEB. In accordance with the manufacturer’s guidelines, the WEBs were delivered through an appropriate size VIA microcatheter (Sequent Medical, Aliso Viejo, California, USA). After the interventions, all elective patients were followed-up in the hospital to detect complications in the neurosurgical recovery unit at least overnight.

### Antiplatelet medication

In accordance with the position of the WEB in the aneurysm, acetylsalicylic acid (ASA) was to be continued for either two or 6 weeks after the WEB embolization. In cases in which an ancillary stent was inserted, dual antiplatelet therapy was initiated immediately, comprising 10 mg of prasugrel for 6 weeks and ASA for 3 months. The Multiplate® (Accumetrics, San Diego, CA, USA) test was used to determine the platelet response to antiplatelet medication. Multiplate Adenosine Diphosphate <30U was considered a sufficient response.

For cases of aneurysmal subarachnoid hemorrhage, apart from heparin in flushing saline (1000 IU/l), no antiplatelet medication was used.

### Radiological and clinical follow-up

Radiological follow-ups were routinely performed with digital subtraction angiography (DSA) at 6 months and (MRA) at 24 months after the WEB treatment. Decisions on re-treatment for recurrence after the initial WEB treatment were made at the multidisciplinary meeting. The Radiological outcome was reported according to Raymond-Roy (RR) occlusion classification. RR 1 and RR 2 were considered as adequate outcomes, while RR 3 inadequate.

Clinical follow-up was performed in the outpatient clinic at either three or 6 months after the intervention, and clinical outcomes were assessed according to the Modified Rankin Scale (mRS) score.

### Statistical analysis

Categorical variables are presented as frequencies and percentages, whereas continuous data were by means and standard deviations. Categorical variables were assessed with a chi-square test, whereas normally distributed continuous variables with a t-test and non-parametric variables with a Wilcoxon rank sum test. Normality of the data was assessed using Kolmogrov–Smirnov test and visual inspection. Univariate logistic regression was used to assess the predictive value of the factors in predicting favorable angiographic outcome and re-treatment both 6-months and again at 24-months follow-ups. Predictive factors with a *p*-value below 0.1 were then used in a multivariate logistic regression model. Multicollinearity between these factors was assessed and of the factors that had a correlation coefficient above 0.8, the one with the lower *p*-value was included in the final multivariate logistic regression analysis. All statistical analyses were performed with SAS version 9.4 (SAS Institute Inc). A confidence interval of 95% was used to assess the statistical significance of the results.

### Ethical issues

The local institutional review board of the Hospital District of Southwest Finland granted permission for this retrospective registry study (T011/014/18) and waived the need for formal consent.

## Results

### Study population

Data were available for 91 patients (56 females, 62%) at 6 months and 62 of those patients (39 females, 58%) at 24 months. A total of 28 patients were treated for acutely ruptured IAs. Three aneurysms (3%) were partially thrombosed before the WEB treatment. Patient and aneurysm characteristics are summarized in Table 1.

**Table 1.** Patient and aneurysm characteristics.

	6-months follow-up	24-months follow-up
Age	58.7 ± 10.5	57.7 ± 9.9
Proportion of females	62%	63%
Active smoker	22 (25%)	19 (33%)
Hypertension	50 (55%)	35 (56%)
No. of aneurysms	91	62
Location of aneurysm		
ICA	9 (10%)	6 (10%)
MCA	39 (43%)	26 (42%)
Acom	13 (14%)	5 (8%)
Basilar tip	21 (23%)	17 (27%)
ACA	5 (5)	4 (6%)
Vertebrobasilar	5 (5%)	4 (6%)
Mean maximal diameter	7.9 ± 2.8	8.0 ± 2.9
Mean width	6.3 ± 2.2	6.4 ± 2.2
Mean height	6.8 ± 2.7	6.9 ± 2.6
Mean width of neck	4.6 ± 1.3	4.7 ± 1.3
Multi-lobular	23(25%)	16 (26%)
Partially thrombosed	3 (3%)	2 (3%)
Acutely ruptured	28(31%)	14 (23%)
Bifurcation	83(91%)	56 (90%)
Wide-necked	74(81%)	53 (85%)

ICA, internal carotid artery; Acom, anterior communicating artery; ACA, anterior cerebral artery.

### Treatment characteristics

The WEB-SL was used in all cases (*n* = 91). Ancillary devices were used in 6 patients (7%): coils in 3 cases and stents in 3 cases. Deployment of the WEB device was successful in all cases.

### Radiological outcome and re-treatment

**Figure 1.**
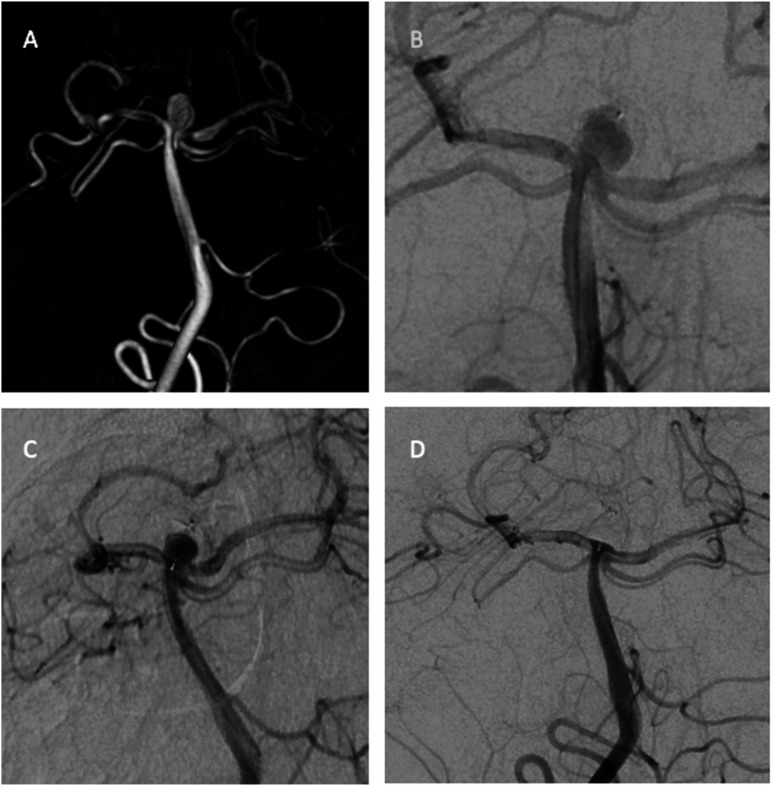
3-D rotational image shows a ruptured basilar tip aneurysm 6 × 4 mm. (a) The immediate postprocedural DSA image shows (b) contrast stagnation in the aneurysm treated with WEB-SL 7 × 3 mm. DSA image (c) shows inadequate occlusion at 6-month follow-up. DSA image shows (d) complete occlusion at 24-month follow-up.

**Figure 2.**
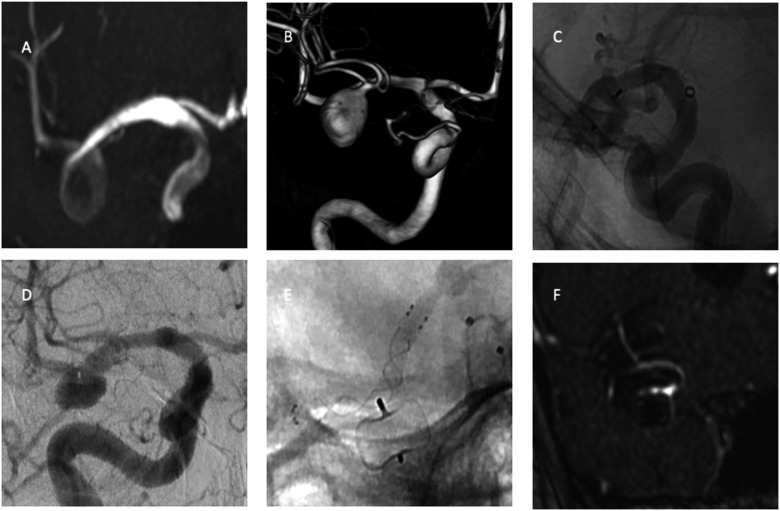
Time-of-flight sequence image (a) shows an unruptured incidental right middle cerebral artery aneurysm. 3-D rotational image (b) shows regular shape 9 × 11 mm aneurysm. The aneurysm is treated with WEB-SL 10 × 7 mm (c). DSA image (d) shows recanalization a year after the WEB treatment. The recurrent aneurysm is treated with intraluminal flow diversion (e). MRA image shows (f) complete occlusion at 24-month follow-up after flow diversion.

The radiological outcomes of 91 patients were available for the 6-months follow-up and for 62 of those patients for the 24-months follow-up. The radiological outcomes of 9 aneurysms (10%) were assessed only with MRA, and the rest were assessed with DSA and MRA. The complete occlusion rate (RR1) was 62% (*n* = 56/91) at the 6-months follow-up while the neck remnant was observed in 27% (*n* = 25/91), thus the adequate occlusion (RR 1/RR 2) rate was 89% (*n* = 81/91) at the 6-months follow-up. At 24-months follow-up, the rate of complete occlusion was 68% (*n* = 42/62) while the neck remnant was seen in 23% (*n* = 14/62). Thus, the rate of adequate occlusion was 91% (*n* = 56/62) at the 24-months follow-up. The radiological outcomes of 3 aneurysms (5%, *n* = 3/62) declined from RR 1/RR 2 to RR 3 between the follow-up visits. Among the aneurysms treated with ancillary devices (*n* = 6), 3 aneurysms showed unfavorable radiological outcomes. A total of 13 aneurysms (14%, *n* = 13/91) retreated during the study period between 2013 and 2020. The treatment methods for recanalized aneurysms after the WEB treatment were simple coiling in 3, stent-assisted coiling in 2, intrasaccular flow diversion with WEB in 4, and intraluminal flow diversion in 4 aneurysms. Radiological outcomes are summarized in Table 2 and illustrative cases in Figure 1 and 2.

**Table 2.** Treatment characteristics and radiological outcomes.

	6-months follow-up		24-months follow-up
WEB SL	91 (100%)		62 (100%)
Ancillary devices			
Coils	3		3
Stents	3		3
Radiological outcomes			
RR 1	56 (62%)		42 (68%)
RR 2	25 (27%)		14 (23%)
RR 3	10 (11%)		6 (9%)

SL, single layer; RR; Raymond and Roy occlusion classification.

### Clinical outcome

The treatment-related morbidity rate was 4% (*n* = 4/91), and one patient (1%) died within a year due to the complications of an ischemic stroke related to the WEB treatment. Favorable outcomes (mRS ≤2) were achieved by 62/63 patients (98%) in the subgroup with unruptured aneurysms and 20/28 patients (71%) in the subgroup with ruptured aneurysms.

### Factors predicting favorable angiographic outcome

In the univariate analysis, the only predictor for inadequate occlusion at the 6-months follow-up was the lobular shape of an aneurysm (odds ratio [OR] 5.6; 95% CI 1.4–22.2; *p* = .01). The aneurysm’s height (OR 2; 95% CI 1.1–3.7; *p* = .02), maximal diameter (OR 1.6; 95% CI 1.2–2.3; *p* = .001), width (OR 1.7; 95% CI 1.2–2.4; *p* = .002), ASR (OR 4; 95% CI 1–15.2; *p* = .03), DNR (OR 7.2; 95% CI 1.1–44.4; *p* = .04), and lobular shape (OR 7.3; 95% CI 1.1–44; *p* = .03) were predictive factors for inadequate occlusion at 24 months. All the thrombosed aneurysms (*n* = 3) showed unfavorable radiological outcomes and required re-treatment. In the multivariate analysis, lobular shape was the only statistically significant factor predicting outcome at 6 months (OR 6.2; 95% CI 1.2–31.7; *p* = .02). However, in the multivariate analysis, no variables were statistically significant for the radiological outcomes at 24 months. None of the patient-related factors (i.e., smoking status, hypertension, age, and sex) affected the radiological outcomes. The results of the analysis of patient- and aneurysm-related factors are summarized in Table 3.

**Table 3.** Factors influencing radiological outcomes.

		6 months			24 months		
		Adequate outcome	Inadequate outcome	*p*-value	Adequate outcome	Inadequate outcome	*p*-value
Height of aneurysm		6.7 ± 2.5	8.3 ± 4.3	>.05	6.4 ± 1.9	11.2 ± 4.6	.02
Width of aneurysm		6.3 ± 2.2	7.1 ± 2.9	>.05	6.1 ± 1.8	9.7 ± 2.9	.002
ASR		1.5 ± 0.5	1.7 ± 0.9	>.05	1.5 ± 0.5	1.9 ± 0.8	.03
DNR		1.4 ± 0.4	1.4 ± 0.5	>.05	1.3 ± 0.4	1.7 ± 0.5	.04
Maximum diameter		7.8 ± 2.6	9.4 ± 4.6	>.05	7.1 ± 2.1	9.1 ± 3.5	.001
Sex				>.05			>.05
Male		29/35	6/35		21/23	2/23
Female		52/56	4/56		35/39	4/39	
Active smoking				>.05			>.05
No		58/65	7/65		37/39	2/39	
Yes		19/22	3/22		16/19	3/19	
Hypertension				>.05			>.05
No		35/41	1/7		25/27	2/27	
Yes		46/50	4/50		31/35	4/35	
Location of aneurysm				>.05			>.05
ICA		8/9	1/9		6/6	0/6	
MCA		37/39	2/39		23/26	3/26	
Acom		10/13	3/13		4/5	1/5	
Basilar tip		19/21	2/21		16/17	1/17	
ACA		5/5	0/5		4/4	0/4	
Vertebrobasilar		2/4	1/2		3/4	1/4	
Acutely ruptured				>.05			>.05
No		54/63	9/63		43/48	5/48	
Yes		27/28	1/28		13/14	1/14	
Location of aneurysm				>.05			>.05
AC		59/66	7/66		38/42	4/42	
PC		22/25	3/25		18/20	2/20	
Wide-necked				>.05			>.05
No		16/17	1/17		9/9	0/9	
Yes		65/74	9/74		47/53	6/53	
Thrombosed aneurysm				>.05			>.05
No		79/88	9/88		56/60	4/60	
Yes		2/3	1/3		0/2	2/2	
**Lobular** shaped				.01			.03
No		64/68	4/68		44/46	2/46	
Yes		17/23	6/23		12/16	4/16	
Bifurcation/Sidewall				>.05			>.05
Bifurcation		75/83	8/83		51/56	5/56	
Sidewall		6/8	2/8		5/6	1/6	
Parent artery leaving from neck				>.05			>.05
No		71/80	9/80		49/54	5/54	
Yes		10/11	1/11		7/8	1/8	

ASR, aspect ratio; DNR, dome-to-neck ratio.

## Discussion

### Radiological outcomes and re-treatment

We investigated the short- and mid-term radiological outcomes of IAs treated with a WEB. In our study, the adequate occlusion rates were 89% and 91% at the 6 and 24 months, respectively. We also found several factors that affected radiological outcomes at 6 and 24 months after WEB treatment.

The WEB has been a game changer for the treatment of wide-necked IAs, which have been challenging to treat with conventional endovascular methods. Since 2011, several studies on the WEB’s safety and efficacy have been published. In our study, the adequate occlusion rates were 89% at 6 months and 91% at 24 months. Kabbasch et al. reported a rate of 84% adequate occlusion at 6 months after WEB treatment.^15^ In a report of three European clinical trials of WEB by Pierot et al., where WEB-DL, - SL, and -SLS were used, the overall adequate occlusion rate at 24 months was 81% (*n* = 98/121).^13^ In our study, all the WEB devices used were new-generation WEB-SL devices, which can provide many advantages over the first-generation WEB-DL devices, such as easy trackability and control.^13^ However, in the sub analysis of three European clinical trial, the similar rate of the adequate occlusion were achieved with both generation of devices (WEB-SL/SLS: 83%; WEB:DL 79%) at 24 months.^13^

Unfavorable outcomes at 6 and 24 months after the WEB treatment were observed in 11% and 9% of the aneurysms, respectively, in our study. Of the inadequately occluded aneurysms at 6 months, 6 were lobular-shaped, 1 was partially thrombosed, and 1 was large (>10 mm). Among the aneurysms inadequately occluded at 24 months, 4 were lobular-shaped, and 2 were partially thrombosed. In previous studies, partial thrombosis of the aneurysm, increasing aneurysm size, a wide ostium (≥4 mm), and an irregular shape were associated with unfavorable radiological outcomes after WEB treatment.^15–17^

The initial radiological outcome after WEB treatment seems to be relatively stable. However, improvements or declines in radiological outcomes have been reported. In our study, a rate of 5% decline from an adequate to an inadequate radiological outcome between 6 and 24 months was detected. A 6% rate of improvement or decline in radiological outcomes between 12 and 24-months follow-up was reported by Mine et al.,^18^ while Pierot et al. reported a 6% improvement and a 13% decline in radiological outcomes over a similar period.^13^ A decline in radiological outcomes was seen in cases that could indicate that the operator’s experience level a significant predictive factor for stable outcome with WEB as the declines in radiological outcomes were seen in the aneurysms treated in the beginning of our experience with the WEB.

The rate of re-treatment within our study period was 14%. In long-term series, the rate of re-treatment has been reported to be between 9% and 20%.^13,18^ The indications for re-treatment can vary somewhat and are difficult to compare between studies. However, neuroendovascular teams consider retreatments with a low threshold for incompletely occluded previously ruptured IAs because of the risk of rebleeding. Of the aneurysms that retreated in our study, two had previously ruptured. The treatment techniques used for re-treatment were simple coiling, stent-assisted coiling, intrasaccular flow disruption with WEB, and intraluminal flow diversion in our study. Beside endovascular techniques, the microsurgical clipping was an option according to a study by Pierrot et al.^19^

### Clinical outcomes

The rates of treatment-related symptomatic thromboembolic complication and mortality in our study were 4% and 1%, respectively. Other studies and a recent meta-analyze have reported rates of treatment-related thromboembolic complications of between 8% and 12%.^18,20,21^ In a recent meta-analysis that included both ruptured and unruptured IAs treated with a WEB, the mortality rate was 1%.^20^ No rebleeding or bleeding of was observed in our study.

### Factors predicting radiological outcome

Aneurysm- and patient-related factors influencing the radiological outcome of aneurysms treated with a WEB have been reported. Kabbasch et al. found that thrombosed aneurysms were prone to recanalization.^15^ In our study, all the thrombosed aneurysms recanalized within 2 years after the WEB treatment. However, we could not confirm it statistically, possibly due to the small cohort size. Assumably, partially thrombosed IAs are not appropriate proper candidates for WEB treatment. Another aneurysm-related factor affecting the radiological outcome was the irregular shape of the IA. Cognazzo et al. noted that irregularly shaped aneurysms tend to occlude inadequately because the optimal sizing of the WEB is more difficult in the treatment of irregular shaped IAs than regular shaped IAs.^16^ Our study confirms that aneurysms with irregular shapes are prone to inadequate outcomes probably due to same observation by Cognazzo et al. Furthermore, we found, in concordance with previous reports,^15,16,21^ that the aneurysm’s height, maximal diameter, width, ASR, and DNR were factors that influenced the radiological outcome. Although a wide ostium and ruptured status have been found to predict unfavorable outcomes,^16,21^ we could not confirm this in our study. Youssef et al. found an association between smoking and aneurysm recurrence after WEB treatment.^22^ However, in our study, none of the patient-related factors, including smoking status and hypertension, was associated with inadequate occlusion.

### Limitations and strengths

The limitations of our study relate to its retrospective design. Furthermore, our study was conducted on a small cohort from a single-center registry. Nevertheless, the results provide valuable information on factor which affect the radiological outcomes of IAs after WEB embolization.

## Conclusions

The WEB provides favorable occlusion rates and low complications for both ruptured and unruptured IAs. Unfavorable radiological outcomes after WEB treatment may be related to aneurysm morphology and size. Furthermore, partially thrombosed IAs do not seem to be appropriate candidates for WEB treatment. Radiological outcomes were not associated with smoking or wide ostium in our study. Multicenter prospective studies are recommended to address factors that influence long-term aneurysm occlusion.

## Abbreviations

IA: Intracranial aneurysm
WEB: The Woven EndoBridge
RR: Raymond and Roy occlusion classification
